# Feasibility Study of Glucose Concentration Measurement of Aqueous Solution Using Time Domain Reflected Signals

**DOI:** 10.3390/s22031174

**Published:** 2022-02-03

**Authors:** Samira Saeedi, Somayyeh Chammani, Georg Fischer

**Affiliations:** 1 Time-Domain Electromagnetics Laboratory, Faculty of Electrical Engineering, K.N. Toosi University of Technology, Tehran 1631714191, Iran; samirasaeedi@email.kntu.ac.ir; 2 Institute for Electronics Engineering, University of Erlangen, 91058 Nuremberg, Germany; georg.fischer@fau.de

**Keywords:** glucose concentration measurement, time-domain dielectric spectroscopy, microwave dielectric spectroscopy, ultra-wideband dielectric spectroscopy, dispersive medium analysis

## Abstract

Recently, wideband microwave spectroscopy (WBMS) has been applied for material characterization. Blood glucose sensing through microwave spectroscopy is usually done with resonant frequency-domain methods. Time-domain (TD) WBMS is a low-cost and convenient technique that can be used for glucose sensing of the aqueous solution. In this paper, early research for the implementation of a TD dielectric spectroscopy setup for glucose concentration measurement is presented. TD reflected signals from water with different glucose content are calculated using inverse Laplace transform. The proposed setup is a quasi-monostatic setup in which measurements are done with two different devices in the frequency range of 0.1 to 6 GHz to make a comparison between frequency domain (FD) and TD methods. Frequency domain (FD) measurement is performed with VNA and two Vivaldi antennas. Then, TD data is obtained using the transforming option of VNA. Direct TD measurement is operated with a maximum length sequence (m-sequence) transceiver. Measurement and numerical results follow the same trend and show good agreement with each other. A monotonic relation between peaks of TD signals and the corresponding glucose concentration is achieved. The variation of the height of the reflected signal’s peak is 0.00002 and 0.0005 for each 50 mg/dL glucose concentration with FD measurements and direct TD measurements, respectively. The glucose concentration range of 25 mg/dL to 400 mg/dL is investigated, and the worst repeatability of this method is 3.65% for 300 mg/dL.

## 1. Introduction

Diabetes Mellitus (DM) is a chronic, metabolic disease characterized by elevated levels of blood glucose, which leads over time to serious damage to the heart, blood vessels, eyes, kidneys, and nerves. About 422 million people worldwide have diabetes, and 1.6 million deaths are directly attributed to diabetes each year [1]. The normal glucose level in the blood ranges from 72 mg/dL to 108 mg/dL, whereas patients with DM may experience levels as high as 400 mg/dL [2]. Having access to affordable treatment, including insulin, is critical for the survival of diabetics. Therefore, the exact knowledge of the blood glucose level is essential. Those who suffer from diabetes are supposed to measure their blood level several times a day with painful and invasive methods [3]. Currently, blood glucose levels are mostly monitored with ambulatory monitoring devices, where a drop of blood has to be drawn via a lancet and placed onto a chemically pre-treated strip inserted in a reading device [4]. Furthermore, the usual blood glucose monitoring for diabetics consists of three to six measurements per day with glucometers, which are invasive and need at least one drop of blood several times per day [5]. To overcome these problems, precise non-invasive glucose monitoring is in demand.

These days, electromagnetic waves have been used for various medical applications, including blood glucose measurement [2,3,4,5,6,7,8,9,10,11,12], skin cancer [13], and breast cancer [14]. Blood glucose measurement has been researched in different frequency ranges, from radiofrequency [11] to the infrared range [12]. Among methods that have been studied for non-invasive glucose monitoring, microwave spectroscopy (MS) is one of the most popular techniques. Because, as it was mentioned in [15], “in the microwave range, the electromagnetic waves can deeply penetrate the human tissue regions, where the main blood vessels are located”. MS can be divided into two types based on their frequency range, wideband method, and resonant method. In wideband microwave spectroscopy (WBMS), material characteristics are determined by their complex dielectric permittivity in the microwave range. This approach has been proven to be highly valuable since the dielectric property of materials over a broad range of frequencies is unique for each material [16]. Therefore, a relatively straightforward material sensing and characterization can be performed with low to moderate cost integrated microwave radio hardware.

Most of the MS-based glucose-sensing research is based on the resonant method [2,8,9,10,15]. The whole concept of these references is measuring the resonant frequency shift by changing glucose concentration. Moreover, using the quality factor of the resonator instead of resonant frequency is another method for the characterization of the glucose and water solution. In [8,10], detecting glucose levels up to about 270 mg/dL through fingerprints has been proposed using a microwave sensor and vector network analyzer (VNA). Researchers typically first establish sensing schemes for glucose in water and then advance further, studying glucose in the blood. The glucose contents of water were measured in the range of 0 mg/dL to 400 mg/dL in [9], with a sensitivity of 0.008 dB per 100 mg/dL. Moreover, GlucoWise [17] follows an approach of a resonant sensor operating at 58 GHz based on transmission techniques. For this aim, Gouzouasis et al. utilized two patch antennas and VNA for sensing the added glucose content of water [18]. In [18], it has been mentioned that “A change of 0.23 dB in transmission coefficient was achieved per 900 mg/dL glucose variation”. Based on a monotonic relation, this is equivalent to a sensitivity of 0.005 dB for 18 mg/dL change. Since these results have been measured with VNA, a small VNA with a resolution of 0.005 dB will be required to make this sensor portable. In light of further measurement errors, this sensitivity is very weak. Furthermore, performing WBMS using co-planar and planar sensors has been introduced as a suitable approach for detecting glucose variation recently [3,7,19]. In all of these researches, the material under test (MUT) has to be in direct contact with the sensor. Consequently, the sensor’s usage period will be short, which is not financially viable. Furthermore, contact-based measurement can be detrimental to MUT or the sensor in some situations. These issues can be solved by utilizing non-contact MS.

There are two methods for doing WBMS, the frequency domain (FD) and the time-domain (TD). The FD technique usually requires a high-cost and bulky VNA. In contrast with the conventional FD method, TD dielectric spectroscopy is cost, time, and space-efficient. However, some convenient setups were proposed recently in both FD and TD measurements [16,20,21,22,23]. A non-invasive time-domain WBMS system based on an artificial neural network for detecting glucose content of blood was proposed in [7]. A pair of custom-made ultra-wideband (UWB) rectangular patch bio-antennas were placed on a human earlobe, and TD transmission signals with a central frequency of 4.7 GHz were measured using a transceiver [7]. Complex processing and a large duration of measurement are the weaknesses of this system. The proposed transmission setup of [24] is a kind of TD system in the 50 to 67 GHz frequency range. It makes use of Discrete Fourier Transform (DFT) for processing TD transmitted data through the water with 50 mg/dL to 350 mg/dL glucose concentration. However, they just reported the difference between the DFT of different samples without any estimation for glucose concentration. Thus, a monotonic relation is not proven.

The main goal of this study is to compare the FD and TD methods for measuring the glucose concentration of aqueous solutions using WBMS. More precisely, TD transformed from an FD measurement versus a direct TD measurement has been studied. The proposed system is inspired by the previous research in which TD-WBMS systems were presented for milk qualification [25,26]. In contrast to [25,26], in which permittivity of various milk was estimated using fast Fourier transform (FFT), a relationship between the measured reflected TD signal from MUT and its related glucose concentration is sought. First, the TD reflected signal from MUT has been calculated in MATLAB by applying electromagnetic theory equations. Then, the measurements have been conducted in a quasi-monostatic setup with direct FD and direct TD methods using VNA and Ilmsens radar [27], respectively. Water and glucose mixture has been used instead of the blood sample in all the measurements. It is worth mentioning that water has been used in various researches instead of the blood sample [6,9,15,18,19,28,29]. The change of blood plasma permittivity for various glucose concentrations is comparable with that of water for different glucose contents [30]. To the best of the author’s knowledge, there is no TD-WBMS system for sensing the glucose content by processing data in TD. Therefore, a non-contact TD UWB setup for sensing the glucose concentration seems in demand.

The rest of this paper is organized as follows: data on the measured complex permittivity of water with various glucose concentration levels are reported in Section 2; in Section 3, TD reflection is calculated utilizing inverse Laplace Transform; Section 4 introduces the proposed setup for glucose concentration; comparison with previous studies and conclusions are presented in Section 5 and Section 6, respectively.

## 2. Electrical Properties of Aqueous Glucose Solution

Since blood consists of a major amount of water, it can be assumed that its dielectric properties are related to those of aqueous glucose solutions [3]. The Cole–Cole model reported for blood plasma with different glucose concentrations clarifies that the changes in dielectric properties of blood and water are proportional to each other [30]. Therefore, measuring glucose concentration in water can lead to measuring blood glucose concentration in the early stages of research. Such an approach avoids asking for ethical approval from the ethics commission [31] at an early stage of research. The electromagnetic properties of water are required not only for calculating reflected signals from glucose solution but also for analyzing the results of the proposed setup.

**Figure 1:**
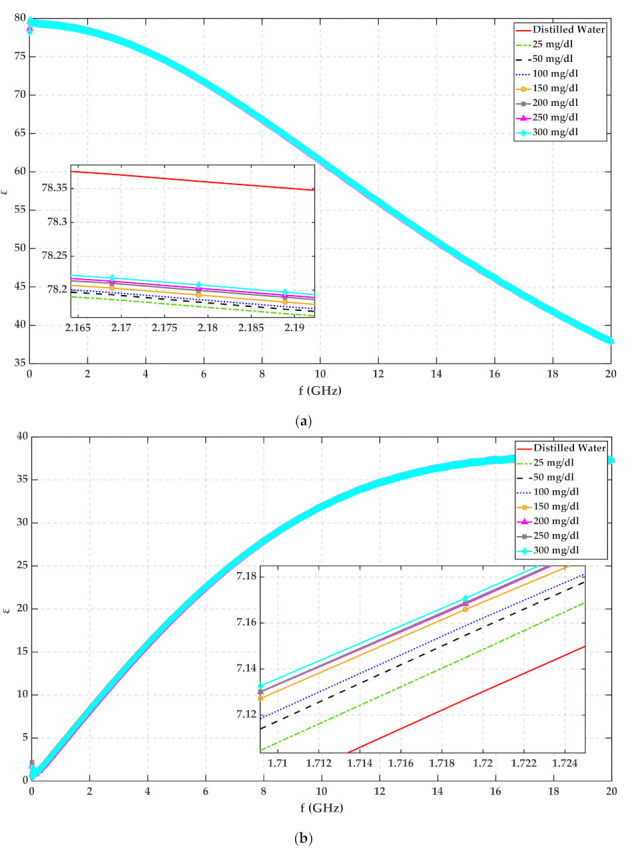
The measured permittivity of 23 °C distilled water with different glucose concentration (**a**) real part (**b**) imaginary part.

The complex permittivity of glucose in water solution has been reported in the literature [3,28]. Although MUT temperature has a critical effect on permittivity, this effect was not considered in [3,28]. Therefore, the complex permittivity of glucose and 23 °C distilled water solution was measured with a commercial coaxial probe and VNA (Agilent N5244A PNA-X) from 10 MHz to 20 GHz frequency band. The measured real and imaginary parts of permittivity for water with different glucose content are plotted in Figure 1.

Water molecules are strongly polar in contrast with glucose with less polarity. Based on Figure 1, the real part of permittivity decreases, and the imaginary part of permittivity increases when we add sugar to water. This phenomenon is just for water. Both $\varepsilon^{\prime }$ and $\varepsilon^{\prime \prime }$ ($\varepsilon^{\prime }$ and $\;\varepsilon^{\prime \prime }$ are real and imaginary parts of permittivity, respectively) increase with increasing the glucose concentration of water and 25 mg/dL glucose solution by the step of 50 mg/dL. It is worth mentioning that this increment for $\varepsilon^{\prime \prime }$ continues till about 18 GHz then $\varepsilon^{\prime \prime }$ follows the opposite trend.

## 3. Reflected Signal Calculation from Debye Medium

The permittivity of water depends on frequency. In [32], it has been mentioned that “The Debye model commonly describes the electrical dispersion behavior of a homogeneous lossy media”. This Model assumes the material dispersion as a linear effect. According to the first order Debye relaxation model, the complex permittivity $\varepsilon_{r}^{*}=\varepsilon^{\prime }-j\varepsilon^{\prime \prime }$ of dispersive materials can be presented with three parameters, as shown in (1). Where $\varepsilon_{s}\;$ and $\varepsilon_{\infty }$ are static and infinite permittivity, and τ is relaxation time [32].

(1) $$\varepsilon_{r}^{*}=\varepsilon_{\infty }+\frac{\varepsilon_{s}-\varepsilon_{\infty }}{1+j\omega \tau }$$

For realizing how TD reflected signals change based on glucose concentration of aqueous solutions, the reflection coefficient ($\Gamma \left(t\right))$ of a Debye half-space in the time domain has been calculated using inverse Laplace transform. Reflection coefficient in Laplace domain can be written as (2) if a TE polarization plane wave $E^{i}\left(t\right)$ is incident on to a dispersive half-space from free space at an incidence angle θ relative to the normal to the interface [33]:

(2) $$\Gamma \left(s\right)=\frac{cos\theta -\sqrt{\varepsilon_{r}\left(s\right)-sin^{2}\theta }}{cos\theta +\sqrt{\varepsilon_{r}\left(s\right)-sin^{2}\theta }}$$

For calculating inverse Laplace of a function, when s→∞ the function must approach zero. However, $\Gamma \left(s\right)$ approaches a non-zero value when s→∞. Therefore, $\bar{\Gamma }\left(s\right)=\Gamma \left(s\right)-\Gamma_{\infty }$ is defined where $\Gamma_{\infty }$ is [33]:

(3) $$\Gamma_{\infty }=\underset{s\to \infty }{\lim}\Gamma \left(s\right)=\frac{1-K_{TE}}{1+K_{TE}}$$

(4) $$K_{TE}=\frac{\sqrt{\varepsilon_{\infty }-sin^{2}\theta }}{cos\theta }$$

**Figure 2:**
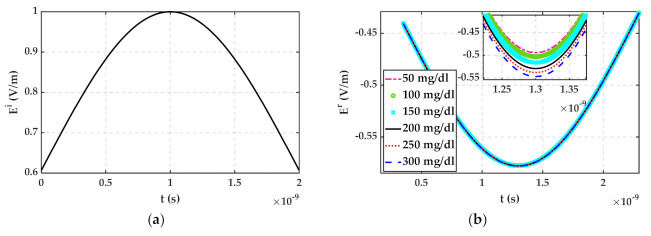
(**a**) Incident field to glucose concentration half-space medium (**b**) Reflected field from different glucose concentration medium.

Rothwell calculated the $\bar{\Gamma }\left(t\right)$ based on (5) [33]:

(5) $$\frac{\bar{\Gamma }\left(t\right)\;}{F_{TE}}=s_{B}g_{1}\left(t\right)-2s_{B}{}^{2}\frac{K_{TE}}{1-K_{TE}{}^{2}}\left[e^{-s_{At}}u\left(t\right)\right]*g_{2}\left(t\right)$$

where

(6) $$g_{1}\left(t\right)=e^{-\frac{t}{\tau }}\left[\left(K_{TE}-1\right)\bar{I_{0}}\left(s_{B}t\right)-\left(K_{TE}+1\right)\bar{I_{1}}\left(s_{B}t\right)\right]u\left(t\right)$$

(7) $$g_{2}\left(t\right)=e^{-\frac{t}{\tau }}\left[\left(K_{TE}-1\right)\bar{I_{0}}\left(s_{B}t\right)+\left(K_{TE}+1\right)\bar{I_{1}}\left(s_{B}t\right)\right]u\left(t\right)$$

(8) $$F_{TE}=\frac{2K_{TE}}{\left(1+K_{TE}\right)\left(1+K_{TE}{}^{2}\right)}$$

(9) $$s_{A}=\frac{1}{\tau }\frac{\varepsilon_{s}-1}{\varepsilon_{\infty }-1}$$

(10) $$s_{B}=\frac{1}{2\tau }\frac{\varepsilon_{s}-\varepsilon_{\infty }}{\varepsilon_{\infty }-sin^{2}\theta }$$

(11) $$\bar{I_{n}}\left(x\right)\equiv e^{-\frac{t}{\tau }}I_{n}\left(x\right)$$

where $I_{n}$ is modified Bessel Function of the first kind and order n. The Debye parameters of glucose and 23 °C distilled water solution have been extracted in MATLAB using plots of Figure 1 and listed in Table 1. Then, the reflected signal $E^{r}\left(t\right)$ according to $E^{r}\left(t\right)=E^{i}\left(t\right)*\Gamma \left(t\right)$ has been calculated in MATLAB for water with 50 mg/dL, 100 mg/dL, and 150 mg/dL glucose when $E^{i}\left(t\right)$ is a Gaussian pulse as shown in Figure 2a. Reflected signals regarding different glucose concentrations have been plotted in Figure 2b.

It can be seen that the depth of reflected signals in Figure 2b decreases with increasing glucose concentration. Although an arbitrary shape dielectric object with a finite thickness plays the role of MUT in the proposed setup, modeling it as a dielectric half-space and ignoring higher-order reflections can be an acceptable assumption. Consider a dielectric object with thickness l≥10 mm, the time difference between first and second wall reflection is $∆t=\frac{2\times l\times \sqrt{\left|\varepsilon_{r}{}^{*}\right|}}{c}$. In the microwave frequency band, $\sqrt{\left|\varepsilon_{r}{}^{*}\right|}$ for different glucose concentrations is more than 7, then ∆t will be larger than 0.46 ns. In a UWB system with more than 2.5 GHz bandwidth, this time difference is large enough for separating the first wall reflection. Water-based solutions are high loss dielectric, and their higher-order reflections can be neglected easily because of their small magnitude. Therefore, the same trend for TD reflected signals from glucose solution is expectable in simulations and measurements.

## 4. Proposed Setup

WBMS can be performed in either quasi-monostatic setup or bistatic setup. Usually, a quasi-monostatic setup requires two adjacent identical antennas for measuring reflected signals from MUT. Reversely, the MUT is placed between two antennas, and the transmission signal is recorded by the receiver in a bistatic method. In this section, a quasi-monostatic setup has been proposed for sensing the glucose concentration of aqueous solutions. The quasi-monostatic spectroscopy has the advantage of smaller required space for the whole system. Moreover, as water is a high loss material, the transmitted signal is weaker than the reflected signal. Therefore, an expensive analog to digital converter (ADC) with a higher resolution will be required for bistatic cases.

First, the setup has been simulated in CST studio suite full-wave software. Then, two different measurement methods have been utilized for proving the feasibility of TD signals for glucose sensing. In the former, FD measurement has been done with VNA and two Vivaldi antennas, and TD signals have been recorded by the transform option of VNA. The second one is a direct TD measurement in which a maximum length sequence (m-sequence) transceiver has been used for sensing MUT. These two setups have been operated in the frequency range of 0.1–6 GHz.

### 4.1. Simulation Results

**Figure 3:**
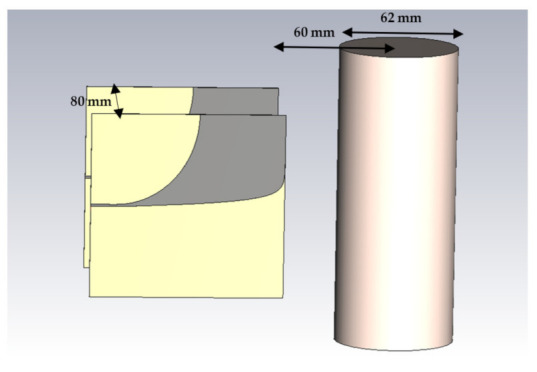
Simulated quasi-monostatic setup.

Figure 3 shows the simulation setup in which transmitter and receiver antennas are two identical Vivaldi antennas. The distance between antennas is 80 mm. To have a compact setup, the required space has been chosen to be as small as possible. The radiative near-field region for an antenna with maximum dimension D at the wavelength of  λ is defined based on (12):

(12) $$0.62\times \sqrt{\frac{D^{3}}{\lambda }}\leq \mathrm{radiative}\;\mathrm{near}-\mathrm{field}\;\mathrm{region}\leq \frac{2\times D^{2}}{\lambda }\;$$

For an antenna with the maximum dimension of 100 mm at 2.9 GHz central frequency, this region will be from 60 mm. Hence, the center of 62 mm diameter MUT is located 60 mm from the antennas. On the other hand, the effect of the surrounding medium will rise with increasing the distance between MUT and antennas.

**Figure 4:**
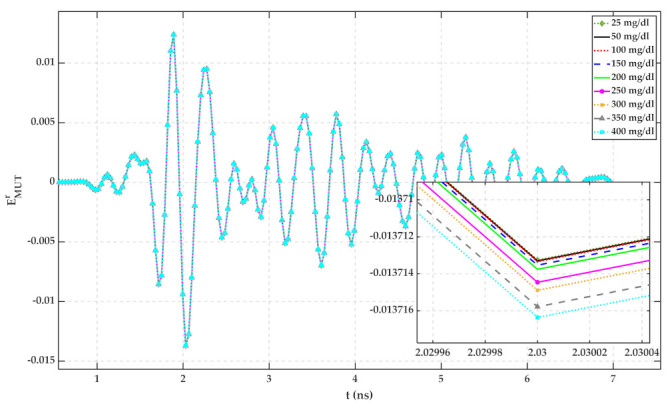
Simulation result for reflected signals from water with the different glucose content.

The container thickness is 2 mm, and its material has been chosen as lossy quartz with a dielectric constant and loss tangent of 3.75 and 0.0004, respectively. Measured complex permittivity of Section 2 has been imported to CST for defining dispersion characteristics of MUTs. The simulation has been operated in the frequency range of 0.1 GHz to 6 GHz. The simulation results for water with different glucose concentrations are plotted in Figure 4.

**Figure 5:**
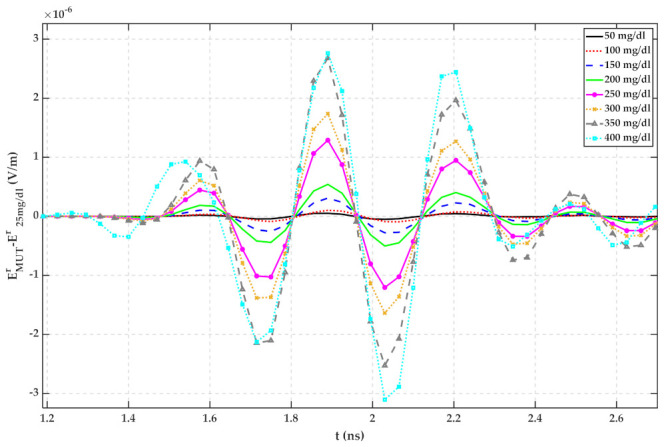
Simulation result for reflected signal differences of various MUTs with respect to water with 25 mg/dL glucose.

Based on Figure 4, the reflected signal increases with increasing glucose content. Hence, it can be seen that the glucose content of water is distinguishable with measuring TD reflected signals. For ignoring the mutual coupling between the antennas and the effect of the container, signals regarding different MUTs are subtracted from signals related to 25 mg/dL and shown in Figure 5. In this paper, water with 25 mg/dL glucose has been utilized as a reference signal because, based on Figure 1, the permittivity of water does not follow the same trend as other samples. In Figure 1b, the imaginary part of the water is close to that of 175 mg/dL. It seems, by adding a small amount of glucose to water, its electrical properties change.

### 4.2. Frequency Domain Measurement Results

**Figure 6:**
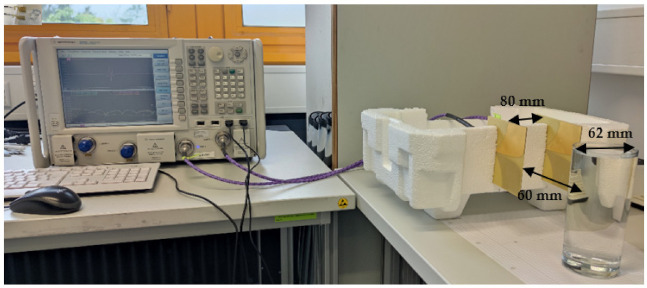
Proposed FD measurement setup.

**Figure 7:**
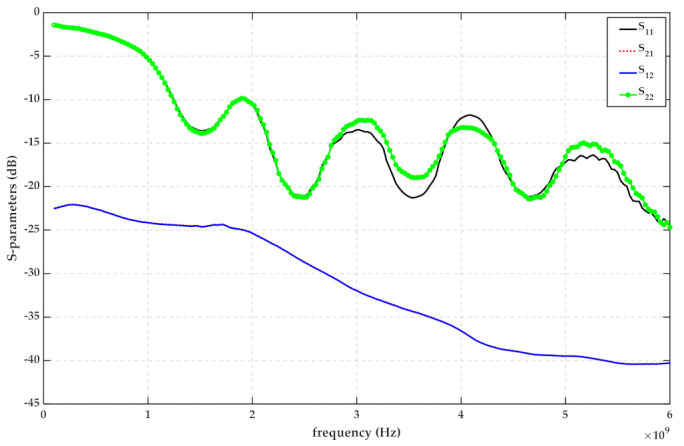
Measured scattering parameters of the empty setup.

The measurement configuration is shown in Figure 6. All the details are the same as with the simulation setup. The measurements of this section have been conducted along with measurements of Section 2 to examine the effect of the electrical properties of the liquid on its reflected signal. The Agilent N5244A PNA-X has been utilized for measuring the S-parameter and transforming them to TD data. First, the measurement has been taken on the empty setup when the container is not in place. The measured scattering parameters of the empty setup for 201 frequency samples have been illustrated in Figure 7. It can be seen that the antenna has an acceptable matching over the desired frequency band of 0.1–6 GHz.

**Figure 8:**
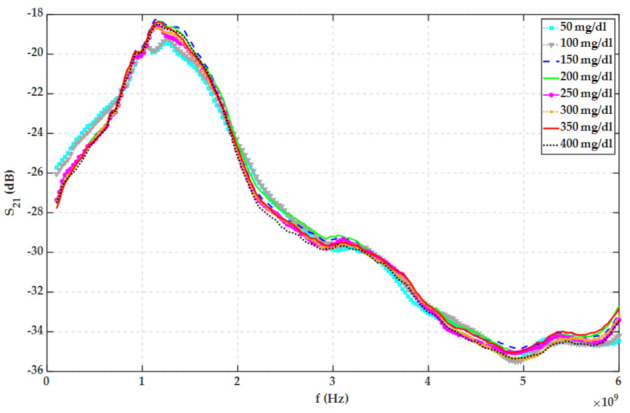
Measured scattering parameters regarding water with the different glucose content.

**Figure 9:**
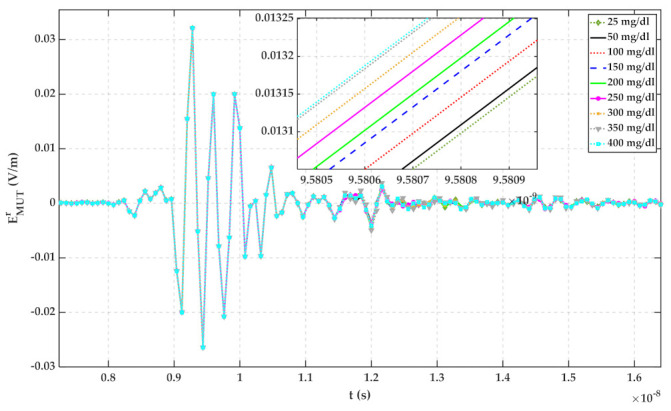
Transformed TD reflected signals regarding different MUTs (measured with PNA-X).

In each step, 200 mg glucose has been weighed with a 1 mg accuracy scale and added to 0.4-L 23 °C distilled water. $S_{21}$ of the bottle full of water and glucose solution has been measured with VNA. Figure 8 shows $S_{21}$ for water with various glucose contents. Based on Figure 8, no feasible relationship has been found between the $S_{21}$ and glucose concentrations on the whole frequency band. The discrete inverse z-transform of $S_{21}$ performed by VNA for various MUTs has been plotted in Figure 9.

**Figure 10:**
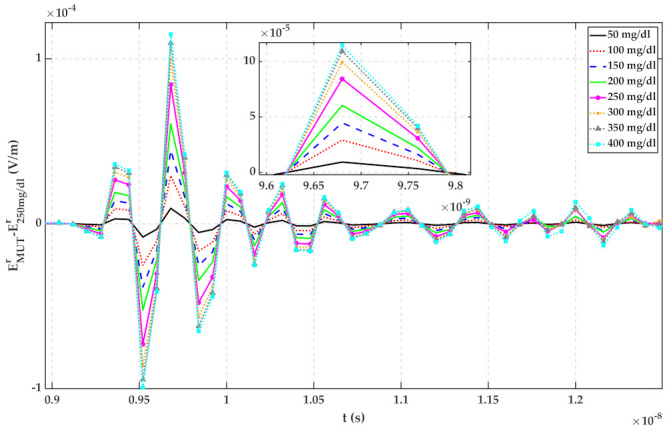
Reflected signal differences of various MUTs concerning water with 25 mg/dL glucose (measured with PNA-X).

According to Figure 9, the more the glucose content of water, the larger the height of the peak of the reflected signal. This can be justified based on the results of Figure 2 and Figure 4. To estimate the glucose concentration of water according to reflected TD signals, the signals regarding water and glucose solutions have been subtracted from the signal obtained from water with 25 mg/dL glucose content and plotted in Figure 10. As can be seen by comparing signals of Figure 9 and Figure 10, the signals up to 9.2 ns are mutual coupling between antennas, and the signals after 11.5 ns are the signals reflected from the wall and other existing things in the laboratory. Therefore, the signals from about 9.8 ns to 11.5 ns can be considered as the response of MUTs to the incident wave.

### 4.3. Time Domain Measurement Results

**Figure 11:**
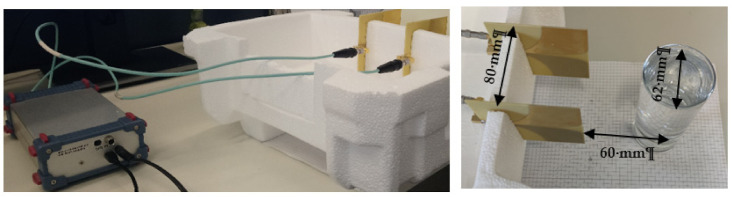
Measurement setup with Ilmsens radar.

In this section, the measurements have been done using Ilmsens radar, as shown in Figure 11, in the frequency range of 0.1–6 GHz. The transmitted power and time resolution of this sensor are about −7 dBm and 78 ps, respectively. All dimensions are the same as in Section 4.2. This radar is a UWB m-sequence transceiver.

**Figure 12:**
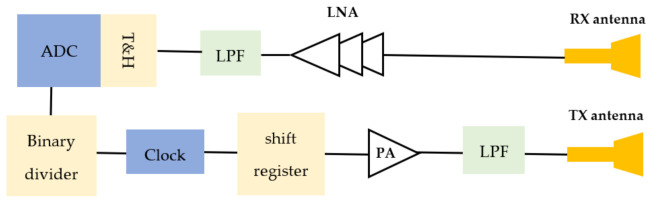
Block diagram of the m-sequence radar.

The block diagram of the m-sequence radar is shown in Figure 12. Typical UWB radars require sub-nanosecond pulse or frequency modulated continuous wave (FMCW) chirps for their operation. Sub-nanosecond pulse can be generated by step recovery diode (SRD). This method suffers from a high peak-to-average power ratio, and the FMCW method has high power consumption and a limited frequency band [34]. To address these problems, an m-sequence transceiver is used. The m-sequence is generated by a digital shift register, which is pushed by a stable RF-clock with frequency $f_{c}$ [34]. The spectral band of $\;\left[0\;f_{c}\right]$ contains 80 percent of the total signal energy of the m-sequence. This drastically reduces the stress caused by strong electric fields, which the MUT would be exposed to due to the small volume of interaction [34].

**Figure 13:**
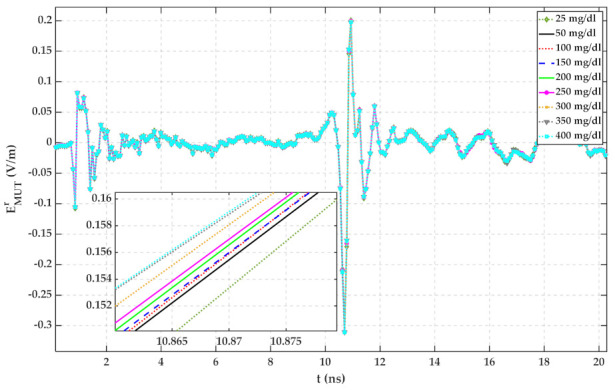
TD reflected signals regarding different MUTs (measured with m-sequence radar).

Same as Section 4.2, the MUT is a mixture of 23 °C distilled water with different amounts of glucose. Reflected signals in the time domain have been recorded by a computer. Each MUT measurement has been repeated 10 times to verify the repeatability of the measurement. The average of reflected signals from each MUT has been calculated and plotted in Figure 13. The signal from up to 10 ns is the internal leakage of the radar.

**Figure 14:**
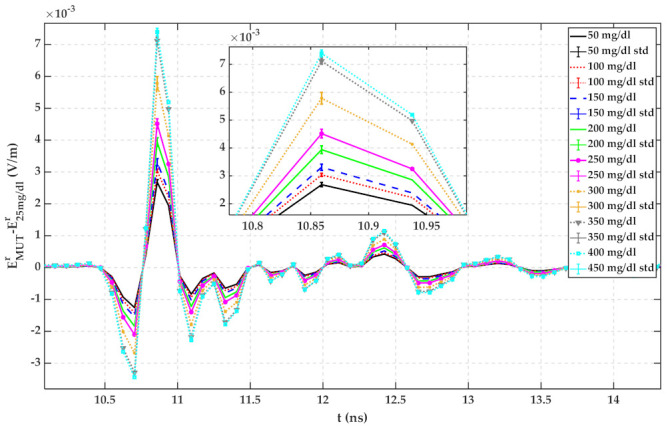
Reflected signal differences of various MUTs concerning water with 25 mg/dL glucose (measured with m-sequence radar).

Plots of Figure 13 have been calculated by subtracting reflected signals of MUTs from water with 25 mg/dL glucose content. The standard deviation (std) of 10 measurements for each MUT has been calculated using (13) and is shown in Figure 14. For a random variable vector A made up of k scalar observations with the mean of μ, the standard deviation is defined as:

(13) $$\mathrm{std}=\sqrt{\frac{1}{k}\overset{k}{\underset{i=1}{\sum }}{\left|A_{i}-\mu \right|}^{2}}\;$$

It can be seen from both figures that the reflected signal regarding more glucose content has a larger peak height.

### 4.4. Mapping the Glucose Concentration of Water to the Maximum of Reflected Signals

**Figure 15:**
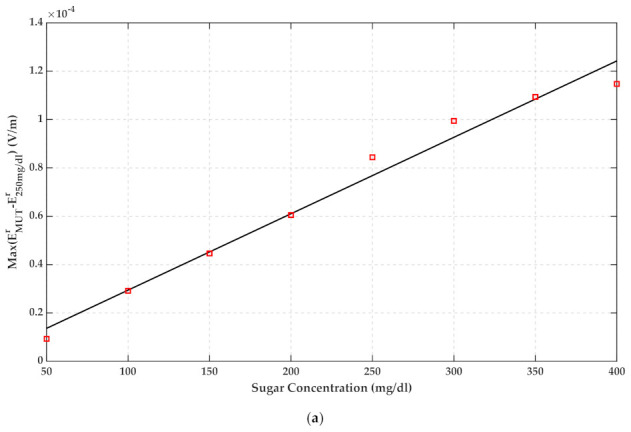

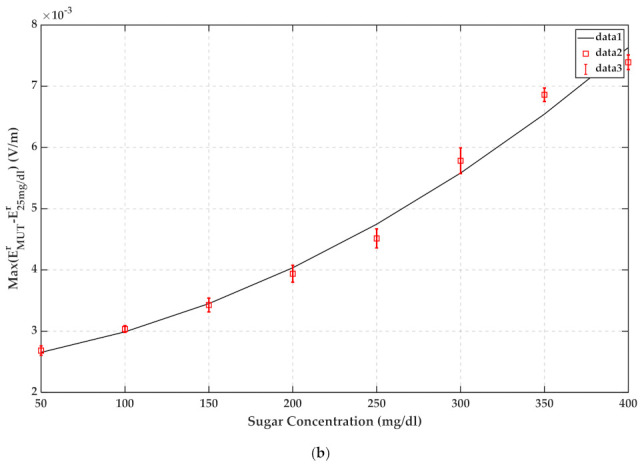
Maximum of reflected signals and corresponding linear fits as a function of glucose concentration (**a**) VNA measurement result (**b**) m-sequence radar measurement result.

For estimating the glucose content of water, a monotonic relation between the height of the peaks of signals in Figure 10 and Figure 14 and the corresponding glucose concentration has been performed and illustrated in Figure 15. The root means square error (RMSE) of both fits is below 0.0017. For example, it can be extracted from Figure 15b if the maximum of the reflected signal was 0.003, the glucose content of water would be 100 mg/dL.

Although this paper is an early step study of this method, the minimum amount of glucose that is detectable using this method is 50 mg/dL. Although 60 mg/dL is commonly cited as the lower limit of normal glucose, symptoms of hypoglycemia usually do not occur until 50 mg/dL [35]. Hence, this limit of detection is acceptable. The operating range for this method is 25 mg/dL to 400 mg/dL. Indeed, based on the measurements, this method can work up to 1000 mg/dL. However, more measurements and statistical data will be needed; that is the purpose of future study. The sensitivity of FD and TD methods are 0.00002 and 0.0005 per 50 mg/dL, respectively. The repeatability of the measurements has been calculated based on (14). The minimum of repeatability is 1.6% for 400 mg/dL, and the maximum is 3.6% for 300 mg/dL.

(14) $$\%\mathrm{Repeatability}=\frac{\mathrm{std}}{\mu }\times 100$$

## 5. Discussion

TD microwave dielectric spectroscopy was shown to be promising for measuring the glucose concentration of the aqueous solution. As mentioned in this paper, although there was no relationship between $S_{21}$ of the various MUTs, the peak heights of the TD signals were mapped to their related glucose content. However, some research reports the change of the S-parameter with glucose concentration using the resonance method, but the sensitivity is weak and needs an accurate bulky VNA. Using TD signals, coupling, multiple reflections, and multipath effects were observed separately from the MUT. Based on this paper, TD dielectric spectroscopy measurement can be done in either the FD or TD method. However, interpreting data regarding the glucose content in TD is much easier. Therefore, if one does the measurement in FD, it is advisable to transform the data to TD for better interpretation. Inverse z-transform is required for calculating TD signals from the measured S-parameter. Nevertheless, TD measurement usually is convenient and has the potential of being portable. An acceptable sensitivity for measuring the glucose content of water is reachable with direct TD measurement, as shown in Section 4.3. Using a water and glucose mixture instead of the blood sample can be a reliable approximation for the early step of research since, according to reflection theory, the magnitude of permittivity of MUT is the only parameter that affects the reflected signal from the medium. The change of blood plasma permittivity for various glucose concentrations is comparable with that of water for different glucose contents.

According to Figure 5, Figure 6, Figure 7, Figure 8, Figure 9, Figure 10, the reflected signal differences are more obvious in the measurement than in the simulation. CST fitted the imported complex permittivity of dispersive materials with the Debye model. However, the Debye model is the approximation of linear dielectric properties of dispersive materials, and in the real world, we may face different challenges for testing aqueous solutions [29]. According to Figure 10, Figure 11, Figure 12, Figure 13, reflected signal differences for m-sequence radar measurement results are much larger than that of VNA measurement. This happened because the m-sequence receiver amplifies the captured signal with a gain of about 15 dB. Furthermore, it is proof of the claim that glucose sensing can be done with a small transceiver with acceptable accuracy.

One may argue that higher-order reflection can affect reflected signals if a small container is used. However, it was mentioned in the previous paper that the second wall reflection does not have any effect on the measurement data for a 6-cm diameter container [26]. Based on Section 3, with a bandwidth of more than 2.5 GHz, the second wall reflection is separable from TD signals if the water is placed in a bottle with more than 1 cm diameter.

Table 2 compares the proposed non-contact wideband TD setup with the previously reported works in the area of glucose concentration measurement. In [7] is a low-cost TD setup in which glucose concentration was measured through the earlobe with a max error of 16 mg/dL. The weaknesses of this setup are complex processing and being time-consuming. Sensing glucose level with an FD resonant method utilizing VNA and coplanar waveguide was presented in [8,10]. These two setups required an accurate and expensive VNA that could detect about 1.34 MHz resonant shift per mg/dL. Moreover, the early research on the Glucowise sensor was presented in [18]. This sensor needs a VNA which can distinguish 0.005 dB change for the magnitude of transmission coefficient per 18 mg/dL glucose variation. In [24], TD measurement was conducted on the water glucose solution. The changes in DFT of TD data were reported for various glucose concentrations. However, there was not a clear estimation for detecting glucose.

## 6. Conclusions

In this paper, early research on TD analysis for detecting glucose concentration of the aqueous solution was presented. A comparison between FD and TD measurement results and their data analysis was presented. Inverse Laplace transform was served for calculating reflected signals from a Debye medium. No matter whether the measurements were done using the FD or TD method, there was a monotonic relationship between the maximum of TD data and their associated glucose concentrations. The change of peak height of the TD reflected signal is about 0.00002 for 50 mg/dL glucose concentration with FD measurements and 0.0005 with direct TD measurements. This magnitude resolution is reachable with a cheap 12 bit analog to digital converter in the receiver part. Measurement results showed good agreement with calculated numerical data in MATLAB. In comparison with the previous studies, this method has the potential of being portable, non-contact, and convenient.

One of the critical factors for characterizing liquids is temperature. Since the permittivity of liquids is sensitive to temperature, it is important to keep the temperature constant. To get more accurate results, doing measurements at a controllable temperature is one of our future study aims. Since this method is an early research study for sensing the glucose content of the blood, the proposed method must be modified in the future for in-vivo cases. Moreover, fabricating a low-cost and portable transceiver is another direction that authors would intend to follow.

## Figures and Tables

**Table 1:** Extracted Debye parameter for 23 °C water with the different glucose content.

Glucose Concentration	$\varepsilon_{s}$	$\varepsilon_{\infty }$	$\tau \;\left(in\;ps\right)$
50 mg/dL	79.28	4.895	8.19
100 mg/dL	79.29	4.897	8.20
150 mg/dL	79.31	4.901	8.23

**Table 2:** Summary and comparison with related work.

Ref.	Setup Properties	Frequency Range	MUT Type	Method	Price
[7]	UWB planar antenna, transceiver	3.23–12 GHz	Human Earlobe	Direct TD	Low
[8]	coplanar waveguide and VNA	4.5 GHz and 7.5 GHz	Finger for 89–262 mg/dL glucose level	Direct FD	High
[10]	coplanar waveguide and VNA	1.95–2.2GHz	Finger for 90–190 mg/dL glucose level	Direct FD	High
[18]	VNA and patch Antenna	58 GHz	Water with 0–278 mg/dL glucose content	Direct FD	High
[24]	mm-wave radar and PC	57–64 GHz	Water with 0–350 mg/dL glucose content	FD transformed from TD	Medium
This work	Transceiver and UWB antenna	1–10 GHz	Water with 25–225 mg/dL glucose content	Direct TD and TD transformed from FD	Low

## Data Availability

The data that support the findings of this study are available on request from authors.

## Abbreviations

WBMS	Wideband microwave spectroscopy
TD	Time-domain
FD	Frequency domain
m-sequence	Maximum length sequence
DM	Diabetes Mellitus
MS	Microwave spectroscopy
VNA	Vector network analyzer
MUT	Material under test
UWB	Ultra-wideband
DFT	Discrete Fourier Transform
FFT	Fast Fourier transform
ADC	Analog to digital converter
FMCW	Frequency modulated continuous wave
SRD	Step recovery diode
std	Standard deviation
RMSE	The root means square error
